# Computational methods for automated center determination in electron diffraction patterns

**DOI:** 10.1107/S1600576726002384

**Published:** 2026-04-30

**Authors:** Pavlina Sikorova, Miroslav Slouf, Tomas Molnar, Vladislav Krzyzanek

**Affiliations:** a Institute of Scientific Instruments Kralovopolska 147 Brno 61200 Czechia; b Faculty of Mechanical Engineering Brno University of Technology Technicka 2896/2 Brno 61669 Czechia; c Institute of Macromolecular Chemistry Heyrovskeho nam. 1888/2 Prague 6 16200 Czechia; d CEITEC BUT Brno University of Technology Purkynova 123 Brno 61200 Czechia; Goa University, India

**Keywords:** four-dimensional scanning transmission electron microscopy, electron diffraction, center detection, center determination, nanocrystals

## Abstract

Accurate center detection in electron diffraction patterns is critical for all subsequent processing of experimental diffractograms. This study presents and compares several automated approaches – maximum intensity detection, phase cross-correlation, autocorrelation-based detection, pseudo-Voigt profile fitting and Hough-transform-based detection – applied to both polycrystalline diffractograms with characteristic diffraction rings and single-crystal diffractograms showing discrete diffraction spots. The methods were evaluated in terms of accuracy, robustness, speed, preprocessing requirements and applicability across diverse materials that produce a variety of diffraction patterns. Our findings provide practical guidance for selecting center detection techniques in automated diffractogram processing workflows, thus facilitating improved data quality and reliability in crystallographic analyses. Phase cross-correlation has been proven to deliver high performance consistently on polycrystalline diffractograms with diffraction rings, while pseudo-Voigt profile fitting is best suited to monocrystal-like diffractograms with discrete diffraction spots. All the above-mentioned algorithms have been implemented in the recent version of our open-source Python package *EDIFF*, which now offers a user-friendly, flexible and fully automated solution for center detection in diffractograms. These algorithms determine the center of the individual two-dimensional diffraction patterns, while the processing of complete three-dimensional electron diffraction or four-dimensional scanning transmission electron microscopy datasets often includes accurate center determination as part of structure refinement workflows.

## Abstract 2

This study evaluates and compares automated methods for center detection in 2D diffractograms. The methods have been implemented in *EDIFF*, an open-source user-friendly Python package for the processing of electron diffraction patterns.

## 1. Introduction

The two most widely used methods in transmission electron microscopy (TEM) are bright-field imaging and electron diffraction (Fultz & Howe, 2002 ▸). The importance of electron diffraction was further amplified by the development of four-dimensional scanning transmission electron microscopy (4D-STEM) methods, which are available in both transmission (Ophus, 2019 ▸) and scanning electron microscopy (4D-STEM-in-SEM; Slouf *et al.*, 2025 ▸). The typical result of an electron diffraction measurement is a 2D image (diffraction pattern) containing the most intense spot at the center (the spot of the primary beam, which may be hidden below a beamstop or weakened due to dynamic diffraction effects in some special cases) and units to thousands of weaker diffraction spots. Single-crystal diffraction patterns show individual diffraction spots, while polycrystalline diffraction patterns show multiple spots forming typical diffraction rings (Fultz & Howe, 2002 ▸). In each case, the typical analysis of a diffraction pattern starts with the accurate location of the primary beam position, even if it is eclipsed by the beamstop. The primary beam position defines the center of the reciprocal lattice (Gard, 1976 ▸) and acts as the reference point for all diffraction spots.

Fully automated accurate location of the primary beam position in an arbitrary electron diffraction pattern may be surprisingly challenging. As a result, we have not found any simple, lightweight, open-source and truly universal software for reliable center determination in electron diffractograms. Historically, computational center detection has evolved from fully manual to partially automated methods. One of the earliest widely used tools, *Process Diffraction* (Lábár, 2000 ▸; Lábár, 2005 ▸), determines the diffractogram center via the manual positioning of a model diffraction ring onto the experimental diffraction pattern, which works well for TEM diffractograms but fails for modern high-throughput 4D-STEM data. These methods were subsequently complemented by algorithms focused on patterns with diffuse diffraction rings, which relied on manual pre-selection of a diffuse peak, sector-based integration and nonlinear least-squares fitting (Lábár & Das, 2017 ▸). Modern packages like *CrysTBox* (Klinger & Jäger, 2015 ▸; Klinger, 2017 ▸) introduced automatic routines for processing diffraction patterns, but *CrystTBox* remains closed source (MATLAB based) and GUI driven (unsuitable for batch processing). More recent open-source comprehensive Python frameworks that are connected with the recent development of 4D-STEM methods, such as *Py4DSTEM* (Savitzky *et al.*, 2019 ▸, 2021 ▸), *LiberTEM* (Clausen *et al.*, 2020 ▸) and *pyxem* (Cautaerts *et al.*, 2022 ▸), have broad­ened the automation possibilities, but their focus on 4D-STEM datasets limits their application to other types of diffractograms. Their primary algorithms for center detection rely on the presence of a strong central spot. For example, *py4DSTEM*’s center finding routine explicitly requires that the central spot is visible and intense, precluding its use for diffractograms with beamstops that are typical of TEM (Savitzky *et al.*, 2021 ▸). Analogously, *pyxem* offers basic center-of-mass utilities, supporting masking and thresholding, but lacks a dedicated general-purpose diffraction pattern center detection module (Cautaerts *et al.*, 2022 ▸). Finally, *LiberTEM* excels at scalable distributed processing of STEM data like the two previously mentioned packages, but it relies on center-of-mass center detection as far as the processing of individual diffractograms is concerned (Clausen *et al.*, 2020 ▸). However, all three of the above-listed packages (*pyxem*, *LiberTEM* and *py4DSTEM*) also contain computationally intensive algorithms that, among other outputs, yield refined center positions for single-crystal diffraction patterns with arbitrary orientation. These algorithms typically operate in three steps: (i) automatic detection of diffraction spots, (ii) matching the detected spots to an expected or precalculated diffraction pattern, and (iii) using the resulting fit for precise determination of pattern parameters, including the position of the central beam. More information on modern software for diffraction pattern processing can be found in Table S1 in the supporting information.

In addition to the software frameworks discussed above, a few special-case focused approaches for center determination exist. In zone-axis single-crystal diffraction, the center can be refined by exploiting the inversion symmetry of ±*g* (Friedel) reflection pairs, whose midpoints coincide with the true reciprocal-space origin (Fultz & Howe, 2002 ▸; Zuo & Spence, 2017 ▸). In ring-based patterns, center and detector distortions such as ellipticity are commonly refined simultaneously by fitting concentric rings or through geometric calibration (Pekin *et al.*, 2017 ▸; Savitzky *et al.*, 2019 ▸). In 3D electron diffraction, also known as electron diffraction tomography or MicroED), the accurate center position is typically refined as a part of unit-cell indexing and subsequent refinement workflows. In 4D-STEM workflows, the center is often determined as part of a global lattice-fitting procedure, as mentioned at the end of the previous paragraph and discussed elsewhere (Savitzky *et al.*, 2019 ▸).

In this work, we implemented and tested a range of promising, easy-to-use, fully automated and universal algorithms for reliable center detection in arbitrary 2D electron diffraction patterns. These algorithms do not require symmetric diffraction patterns or computationally intensive detection and fitting of all diffraction spots. These algorithms have been implemented and integrated into our open-source Python package for processing electron diffraction patterns, which is named *EDIFF* (https://pypi.org/project/ediff). We employed both traditional well established approaches and less common techniques that have not been designed specifically for diffraction center location.

The traditional center-of-mass method estimates the center from the intensity distribution under the assumption that the direct beam or low-angle scattering dominates near the true center (Wang *et al.*, 2022 ▸; Thronsen *et al.*, 2024 ▸). An extension of the center-of-mass method is the fitting of a central spot by means of a two-dimensional pseudo-Voigt function (Xu, 2009 ▸; Zhang *et al.*, 2022 ▸), and the fitted parameters may provide a better estimate of the central peak position (Abbondanza *et al.*, 2021 ▸). Another traditional approach, based on manual center estimation by a three-point method combined with automatic center refinement, is available in our package as a control if some of the fully automated methods should fail (Slouf *et al.*, 2025 ▸). Cross-correlation methods exploit the inherent symmetry of diffraction patterns, as explored by Pekin *et al.* (2017 ▸) and René De Cotret *et al.* (2018 ▸). The Hough transform, specifically in its circle-detection variant, has been applied to detect concentric ring patterns in powder diffraction data (Du & Yang, 2009 ▸; Adatrao & Mittal, 2016 ▸). All six methods (center-of-mass, pseudo-Voigt fitting, manual three-point, autocorrelation, phase cross-correlation and Hough transform) were applied to tens of diffraction patterns from real TEM and 4D-STEM experiments, which yielded both mono- and polycrystalline diffractograms with or without a beamstop. The position of the primary beam could be located in each of the tested diffractograms by at least one of the automatic center detection methods.

## 2. Materials and methods

### 2.1. Experimental electron diffraction patterns

All diffraction patterns analyzed in this study come from experiments conducted in the laboratory of the second and last authors of this article (MS and VK). Measurements were carried out either in a standard transmission electron microscope using selected-area electron diffraction (TEM/SAED) or in a high-resolution scanning electron microscope equipped with an array detector of transmitted electrons by means of the 4D-STEM method [4D-STEM-in-SEM, as described in our previous studies (Slouf *et al.*, 2021*a* ▸, 2021*b* ▸, 2025 ▸)]. For the present analysis, the data were partitioned into two datasets, as summarized in Table 1 ▸.

### 2.2. Software for automatic location of primary beam position

The position of the primary beam is readily identified when no beamstop is used but becomes more challenging when the beamstop obscures the central spot. To handle both scenarios, we have implemented multiple algorithms for primary beam location in our open-source *EDIFF* package (Section 2.2.1), which provides end-to-end processing of electron diffraction patterns. In particular, we have substantially extended the ediff.center module of the package (Section 2.2.2).

#### 2.2.1. *EDIFF* package for processing of electron diffraction patterns

*EDIFF* is an open-source Python package (https://pypi.org/project/ediff). It processes experimental electron diffraction (ED) patterns, computes the theoretical X-ray diffraction (XRD) pattern for a candidate crystal and compares ED with XRD to confirm the crystal structure. The package has evolved from a set of Python scripts (Slouf *et al.*, 2021*a* ▸, 2021*b* ▸) through a standard Python module focused on powder diffractograms (Slouf *et al.*, 2025 ▸) to the current version that supports processing of both polycrystalline and single-crystal diffractograms (as detailed below). The first step towards handling arbitrary diffraction patterns is a more general module for accurate location of the primary beam position, which is the main subject of this work.

#### 2.2.2. ediff.center module for accurate location of primary beam position

**Figure 1:**
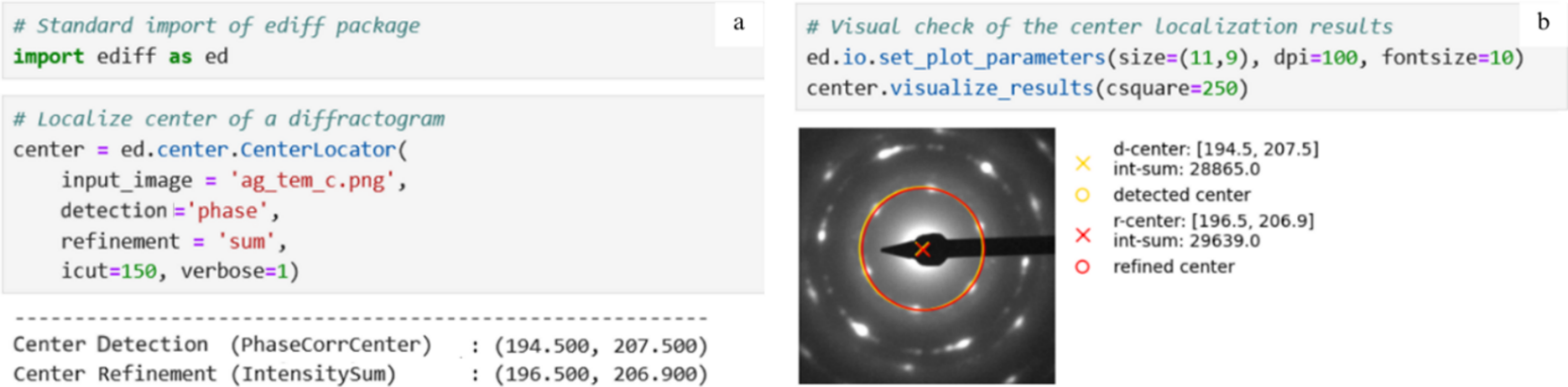
*Jupyter Notebook* interface for the ediff.center module. (*a*) Example code snippet for automated center detection of an ED pattern. The workflow demonstrates how to initialize the CenterLocator, specify the detection method (here phase cross-correlation) and refinement strategy (here intensity sum), and obtain the numerical output for both the initially detected and refined center positions. (*b*) Visualization of the results, showing the diffraction pattern with overlaid markers: the yellow circle and cross mark the detected center, while the red circle and cross indicate the refined center after intensity-based adjustment. Reported coordinates and integrated intensities allow users to evaluate the accuracy of the location.

The ediff.center module (https://mirekslouf.github.io/ediff/docs/pdoc.html/ediff/center.html) focuses on locating the primary beam position. The overall workflow – center location – comprises center detection and center refinement. The module implements six detection algorithms and three refinement algorithms; each algorithm is exposed as a method of the CenterLocator class (Fig. 1 ▸).

The six detection algorithms are summarized in Table 2 ▸. Only the *manual* method requires user input; all others are fully automated. Several center detection methods benefit from operating on edge-enhanced versions of the input image, as reported previously (Pekin *et al.*, 2017 ▸). Accordingly, the ediff.center module includes optional preprocessing using the Sobel edge-detection operator (Pekin *et al.*, 2017 ▸).

The three refinement algorithms are summarized in Table 3 ▸. Again, only the *manual* method requires user input; the other two are fully automated. All three algorithms are based on small shifts of a diffraction ring that passes through a set of strong, approximately equidistant, reflections. The optimized variables are the center coordinates and the ring radius. Fig. 1 ▸ illustrates the approach: rings from the detection and refinement stages are shown in yellow and red, respectively, on the diffractogram.

ED datasets may exhibit elliptical distortion due to external fields or detector imperfections (Hou & Li, 2008 ▸). Although such distortion is typically minimal in most modern devices, the ediff.center module offers a simple correction method that can be applied as part of the center detection step.

#### 2.2.3. Center detection testing workflow

Each method was applied to both test datasets (Table 1 ▸). Reference center coordinates were obtained using the *manual* method followed by automatic refinement (*sum*). All other methods were evaluated without additional refinement to isolate their intrinsic performance. Detection was performed on both raw images and their edge-enhanced counterparts (as described in Section 2.2.2).

After center coordinates had been determined for all images in both datasets, we used another *EDIFF* tool to compute one-dimensional radially averaged intensity profiles from the diffraction patterns – a procedure that is highly sensitive to center location accuracy. Errors in the coordinates directly affect the sharpness, symmetry, peak positions and peak heights of the resulting profiles and thus propagate into subsequent analyses.

Although the width and height of diffraction peaks are primarily governed by the accuracy of center location, image distortions such as the aforementioned ellipticity (Section 2.2.2) may also contribute. However, in modern datasets acquired with calibrated detectors, significant elliptical distortion is uncommon. The data analyzed in this study do not exhibit significant distortions that would affect the comparative evaluation of the tested methods.

### 2.3. Electron diffractogram classification

**Figure 2:**
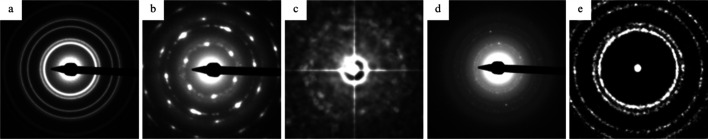
Classification of polycrystalline diffraction patterns. We categorize them on the basis of center detection challenges: (*a*), (*b*) and (*d*) with beamstops, (*b*) broken rings, (*c*) reconstruction artifacts, (*c*) and (*d*) strong noise/background, and (*e*) flat background. Samples: (*a*) and (*e*) Au nano-islands, (*b*) Ag crystals, (*c*) Fe_3_O_4_ nanoclusters, and (*d*) ZnO–CuO nanoparticles.

**Figure 3:**
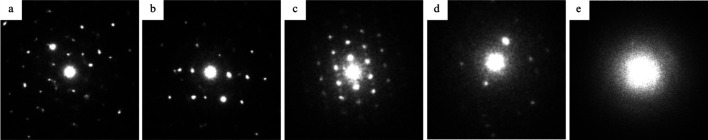
Classification of single-crystal diffraction patterns. We categorize them on the basis of their quality (SNR, background intensity), sorted from (*a*) best quality to (*e*) worst quality. Samples: (*a*) and (*b*) Au nano-islands, (*c*) TbF_3_ nanocrystalline aggregates, (*d*) Fe_3_O_4_ nanoclusters, and (*e*) LaF_3_ nanoparticles in amorphous biological tissue.

We divided the dataset into polycrystalline (Fig. 2 ▸) and single-crystal (Fig. 3 ▸) diffractograms. The polycrystalline diffraction patterns show characteristic rings, while the single-crystal diffraction patterns show diffraction spots. Within each group, every pattern was annotated using morphology- and artifact-based metrics derived from radial and azimuthal intensity distributions, for example ring completeness, eccentricity, spot density and signal-to-noise ratio (SNR). The resulting heterogeneity precluded a single uniform processing pipeline; accordingly, all subsequent analyses are stratified by class to ensure a robust and comparable evaluation. Representative examples of each category are shown in Fig. 2 ▸, Fig. 3 ▸ and Table 4 ▸.

There is a specific case of single-crystal diffraction patterns with a beamstop. They come from traditional transmission electron microscopes where a beamstop was present to protect the camera for both single-crystal and polycrystalline diffractograms. With the advent of modern pixelated detectors, the dynamic range is sufficient to record the central beam without the need for a beamstop. Nevertheless, for completeness, the performance of the center-detection methods is also demonstrated on single-crystal data containing a beamstop (Fig. S1).

Importantly, diffraction patterns cannot be classified solely on the basis of the sample material. The difficulty of diffraction pattern processing depends on multiple factors, as described above. While different materials may statistically predispose diffraction patterns to stronger or weaker scattering under given experimental conditions, the classification used here is based on the actual properties of the recorded diffraction patterns and on the characteristics of the resulting reconstructed powder diffractograms.

## 3. Results

### 3.1. Center detection in polycrystalline diffraction patterns

In polycrystalline diffraction patterns, automated center detection tends to be easy and reliable. Polycrystalline diffraction patterns exhibit approximate centrosymmetry due to concentric Debye–Scherrer rings. This centrosymmetry, which is absent from the great majority of single-crystal diffraction patterns, provides strong constraints for automated center detection.

For all polycrystalline diffractograms, the *manual* center detection method (Table 2 ▸), followed by refinement with the *sum* method (Table 3 ▸), yielded the center coordinates, which we then adopted as the reference standard. We selected this protocol for its simplicity, reliability and direct user control. In addition, the subsequent automatic refinement mitigates possible user-placement errors introduced during the manual step. Finally, the *manual* method itself is not included among the fully automated approaches under comparison.

**Figure 4:**
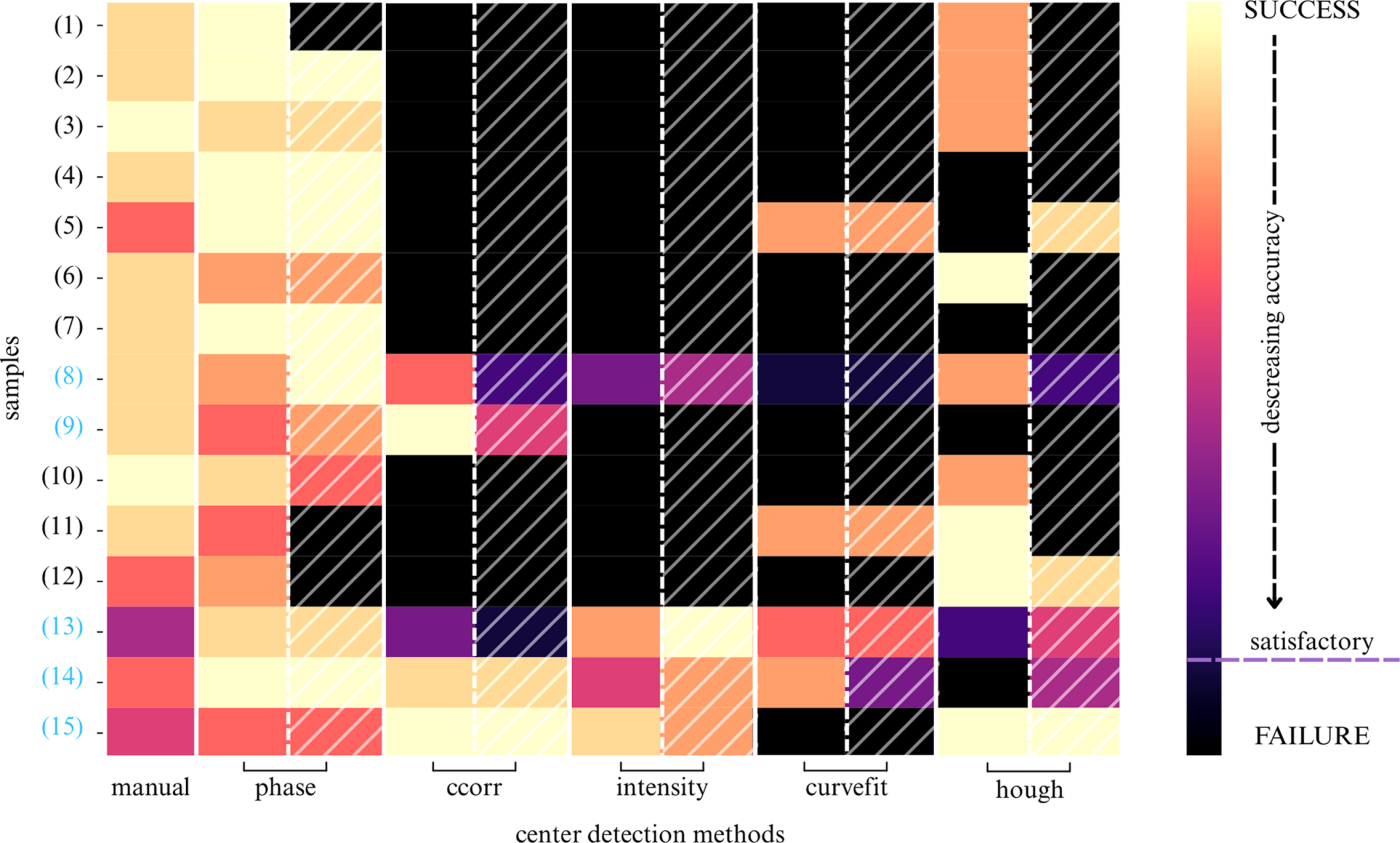
Heatmap illustrating the performance of center detection methods on the polycrystalline dataset D1. Each row corresponds to a diffractogram (numbered according to Table S2; blue-highlighted samples 8, 9, 13, 14 and 15 are from 4D-STEM-in-SEM, while the rest are from TEM) and each column represents a center detection method (*manual* = three-point detection, *phase* = phase cross-correlation, *ccorr* = autocorrelation, *intensity* = maximum intensity detection, *curvefit* = pseudo-Voigt profile fitting and *hough* = Hough transform). Hatched columns indicate the method’s performance on pre-processed diffractograms (edge detection using a Sobel operator). The color bar on the right indicates the performance scale: the lightest color (yellow) indicates the most accurate detection, while the darkest color (black) signifies failure or substantial inaccuracy in center location.

**Figure 5:**
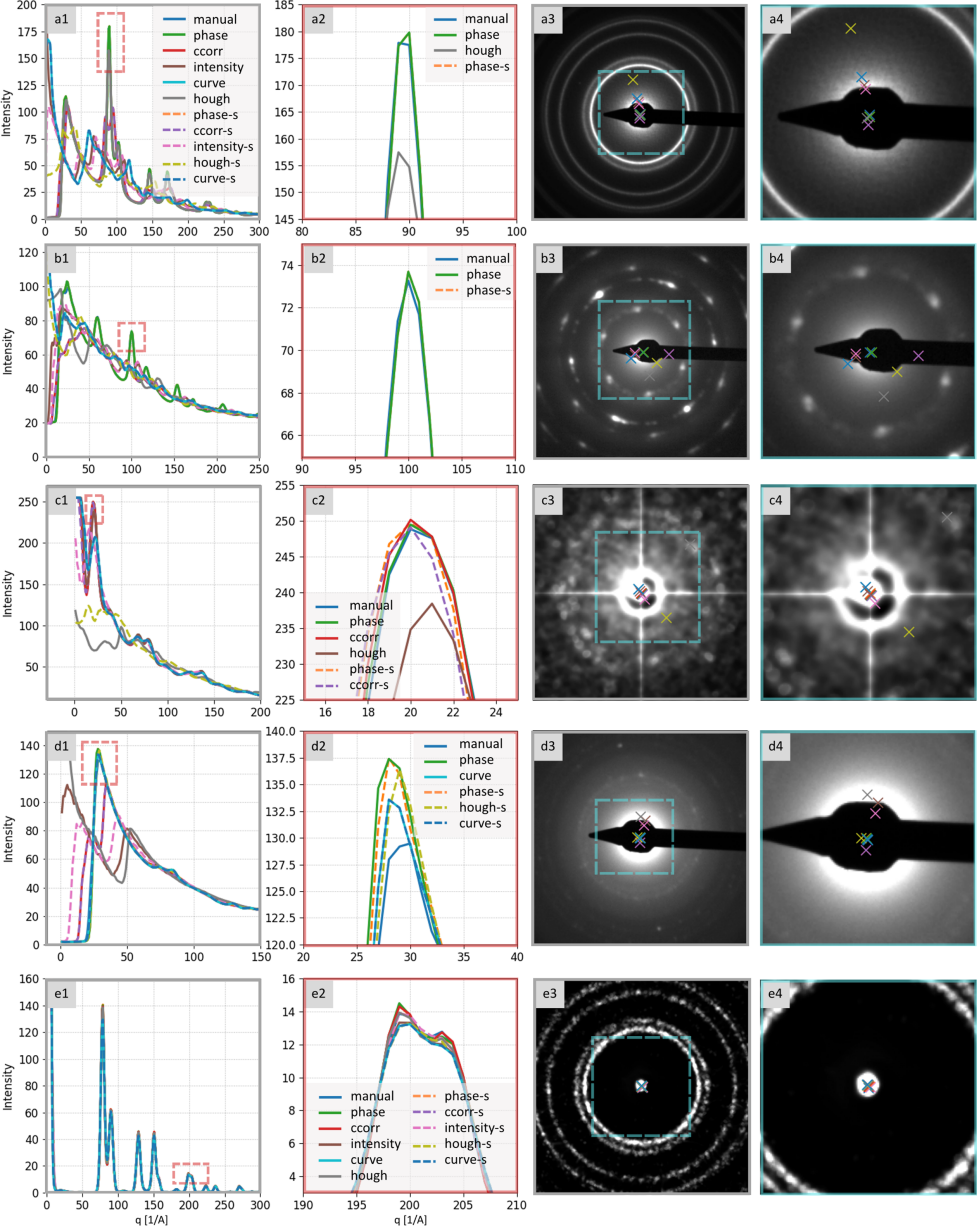
Performance of center detection methods on representative polycrystalline diffractograms corresponding to Fig. 2. Column 1: 1D radially averaged profiles computed using each method’s center (overlaid, color coded). Column 2: enlargement of the dominant peak (red box in Column 1), showing the top-performing method(s). Column 3: original diffractogram with detected centers (color-coded crosses). Column 4: enlargement of the central region (blue box in Column 3) around the direct (primary) beam and the detections. Samples: (*a*) and (*e*) Au nano-islands, (*b*) Ag crystals, (*c*) Fe_3_O_4_ nanoclusters, and (*d*) ZnO–CuO nanoparticles.

The comparison of the performance of all six center detection algorithms is summarized in Fig. 4 ▸. The *phase* center detection method (Section S1.2.3) emerged as the most robust, accurate and generalizable. It performed well regardless of the presence of the beamstop, delivered sub-pixel accuracy and outperformed the reference method in approximately 67% of cases, as shown by sharper and higher peaks in the resulting 1D profiles (Fig. 5 ▸).

Three methods – *intensity*, *ccorr* and *curvefit* – worked well if the primary beam spot was present (samples 8, 9, 13, 14 and 15 in Fig. 4 ▸), but they failed when the primary beam was blocked by a beamstop [samples (*a*), (*b*) and (*d*) in Fig. 2 ▸]. The *hough* method showed intermediate performance – superior to the three above-mentioned methods relying on the primary beam spot, yet still insufficiently robust, as it remained vulnerable to diffractogram asymmetry introduced by the beamstop.

Regarding pre-processing, generic edge detection provided no consistent benefit, except in the case of the *hough* method, where it proved almost essential (see the differences highlighted by the hatched columns in the heatmap in Fig. 4 ▸, or the corresponding 1D profiles labeled with an ‘-s’ suffix in the legend in Fig. 5 ▸). In our tests, the Canny edge detector outperformed the Sobel operator for polycrystalline diffractograms.

### 3.2. Center detection in single-crystal diffraction patterns

Center detection in single-crystal diffraction patterns proved more challenging than in polycrystalline diffractograms. Monocrystalline patterns often lack strong radial symmetry and contain fewer dominant features, reducing the number of reliable cues available to automatic location algorithms.

For all diffractograms, the *intensity* center detection method (Table 2 ▸) followed by refinement with the *manual* method (Table 3 ▸) yielded the center coordinates which we adopted as the reference standard. Unlike the powder diffractograms in Section 3.1, automated refinement by means of the *sum* method could not be used for single-crystal patterns because it assumes a concentric near-symmetric intensity distribution about the true center – a hallmark of polycrystalline rings but not of single-crystal spot patterns. Likewise, *manual* detection which exploits continuous diffraction rings was inapplicable here, as single-crystal patterns lack sufficient spots (≥3) at identical radii.

**Figure 6:**
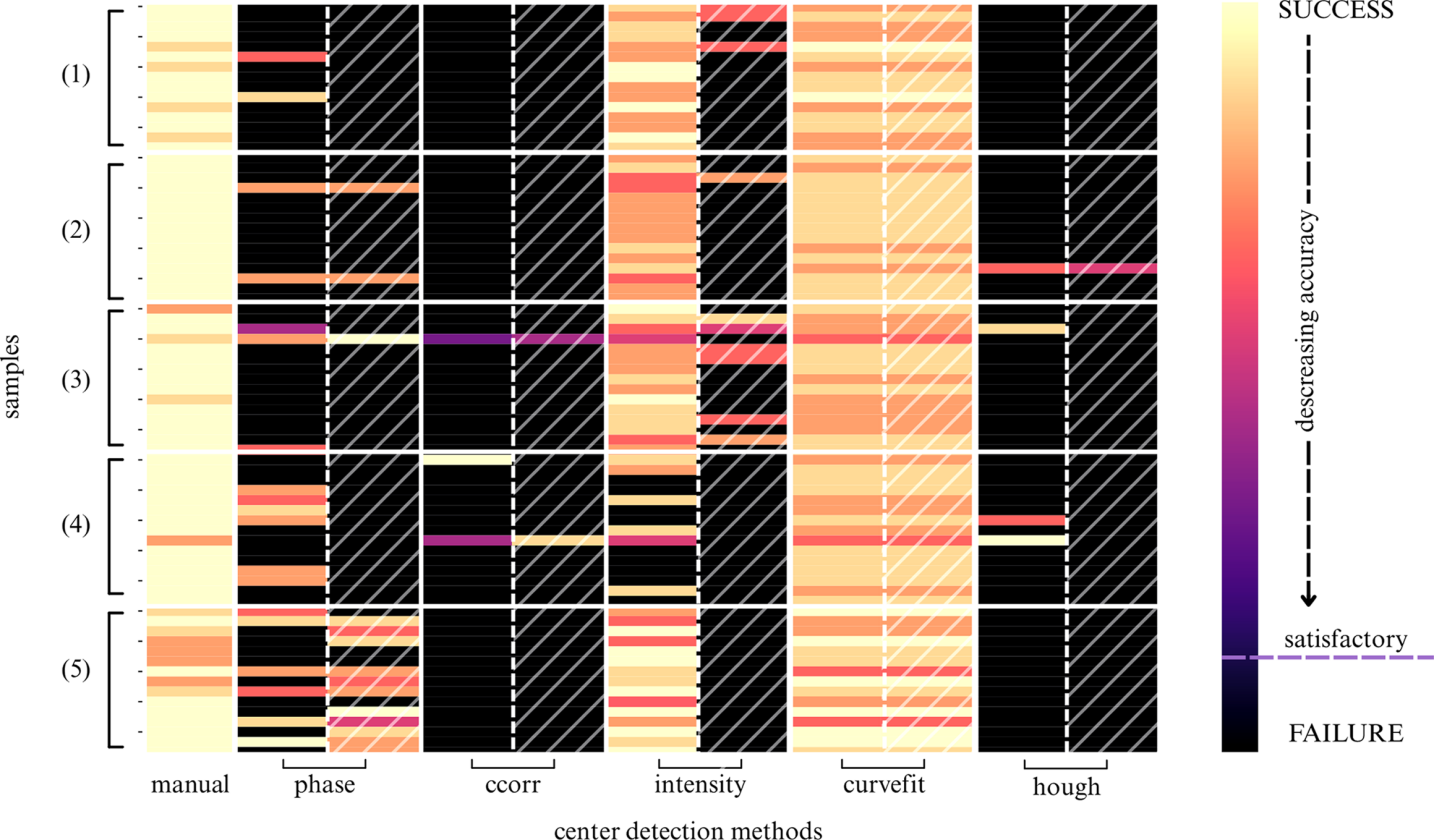
Heatmap illustrating the performance of center detection methods on the single-crystal dataset D2. Each row corresponds to an individual diffractogram [15 patterns per material: (1) Au, (2) TbF_3_, (3) Fe_3_O_4_, (4) LaF_3_ and (5) GdF_3_]. Each column represents a center detection method (*manual* = three-point detection, *phase* = phase cross-correlation, *ccorr* = autocorrelation, *intensity* = maximum intensity detection, *curvefit* = pseudo-Voigt profile fitting, *hough* = Hough transform). Hatched columns indicate a method’s performance on pre-processed diffractograms (edge detection using a Sobel operator). The color bar on the right indicates the performance scale: the lightest color (yellow) indicates the most accurate detection, while the darkest color (black) signifies failure or substantial inaccuracy in center location. The groups (1)–(5) correspond to various diffractogram qualities, as explained in Section 2.3, Table 4 and Table S3. The quality is as follows: (1) perfect, (2) good, (3) bad, (4) poor and (5) intermediate.

The heatmap in Fig. 6 ▸ shows substantial performance variability; no automatic method outperformed the reference approach on the single-crystal dataset, so manual centering remained necessary for optimal accuracy on individual patterns. For large-scale datasets (*e.g.* 4D-STEM-in-SEM) automation is still essential; in our 4D-STEM/powder nanobeam diffraction (NBD) pipeline, patterns unsuitable for reconstruction are filtered automatically and several decision criteria depend on the detected center (Slouf *et al.*, 2021*a* ▸).

**Figure 7:**
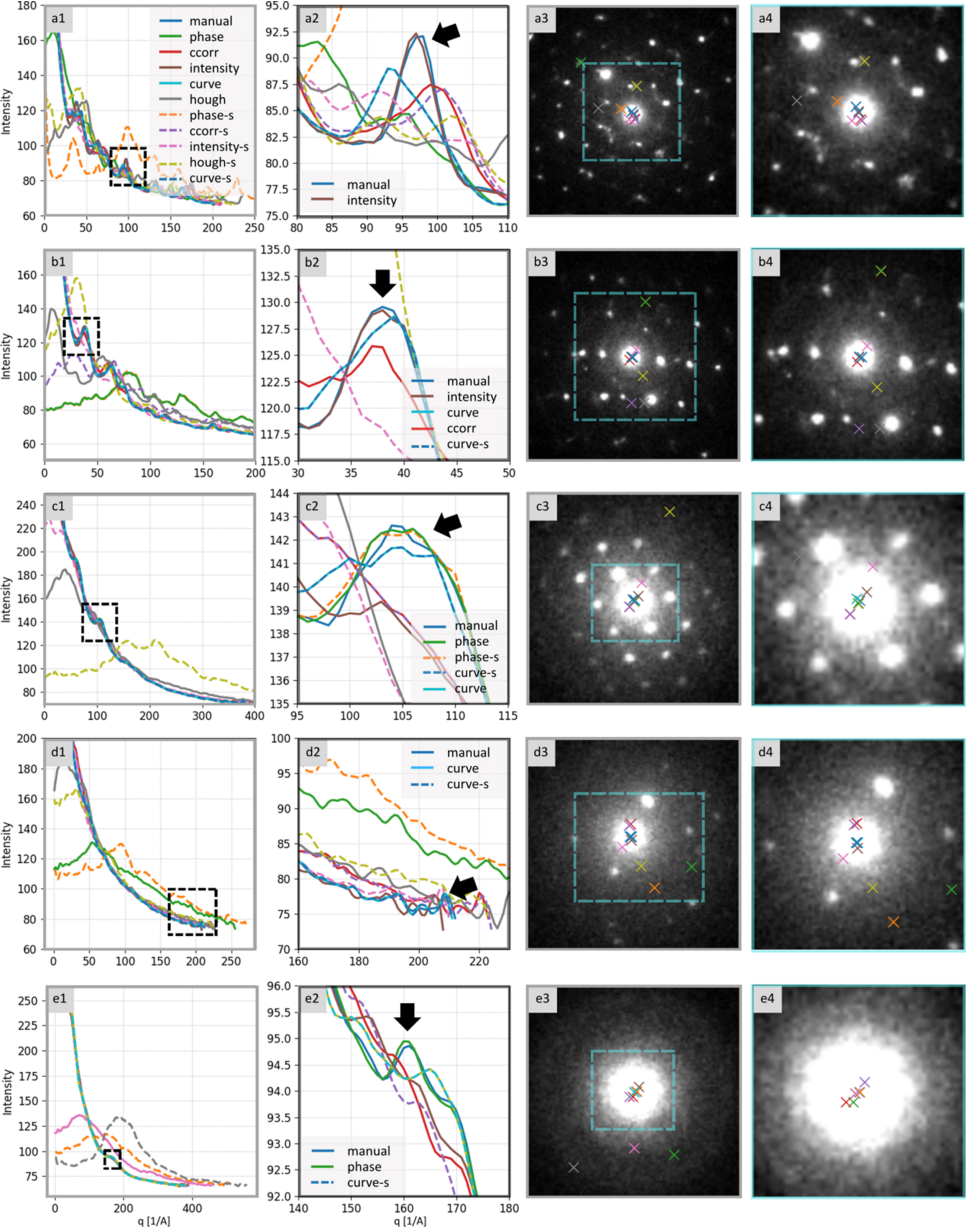
Performance of center detection methods on representative single-crystal diffractograms corresponding to Fig. 3. Column 1: 1D radially averaged profiles computed using each method’s center (overlaid, color coded). Column 2: enlargement of the dominant peak (black box in Column 1), marked with black arrows, showing the top-performing method(s). Column 3: original diffractogram with detected centers (color-coded crosses). Column 4: enlargement of the central region (blue box in Column 3) around the direct (primary) beam and the detections. Samples: (*a*) and (*b*) Au nano-islands, (*c*) TbF_3_ nanocrystalline aggregates, (*d*) Fe_3_O_4_ nanoclusters, and (*e*) LaF_3_ nanoparticles in amorphous biological tissue.

Method-wise, the *phase* detector underperformed on the single-crystal dataset, *ccorr* showed near-total failure across individual patterns, *hough* failed entirely and edge-detection preprocessing did not measurably improve correlation-based methods. By contrast, *intensity* and *curvefit* were the most effective. The *curvefit* method succeeded across all samples (as indicated by the absence of dark rows in the columns corresponding to this method in the heatmap in Fig. 6 ▸). The *intensity* approach was less reliable for low-quality LaF_3_ patterns, where no clear Bragg peaks were visible beyond the saturated central beam (note several dark rows in the intensity column for the LaF_3_ sample in Fig. 6 ▸). The performance of center detection methods on representative single-crystal diffractograms is shown in Fig. 7 ▸.

Examples of single-crystal diffraction patterns with beamstops (Fig. S1), along with the performance of the corresponding center detection methods, are provided in the supporting information (Section S1.3).

### 3.3. Computational efficiency

The computational efficiency of all methods was benchmarked for speed (Table S4). The *intensity* method was the fastest, locating the center within hundredths of a second per pattern, whereas the *phase* method was slower but consistently delivered the highest accuracy. The *curvefit* method exhibited dataset-dependent runtimes: on the polycrystalline dataset it was relatively fast, while on the single-crystal diffractograms it was the slowest. Edge-detection preprocessing using the Sobel operator had minimal impact on the runtime of most methods. By contrast, combining the Hough detector with Canny edge preprocessing substantially increased runtime.

## 4. Discussion

### 4.1. Diffractogram processing challenges

#### 4.1.1. Polycrystalline diffractograms

Guided by the classification in Section 3.1 (Fig. 2 ▸, Fig. 3 ▸ and Table 4 ▸), we identified four recurrent challenges that impede robust automated center detection in polycrystalline diffractograms exhibiting Debye–Scherrer rings:

(i) Beamstop blocked (BB) diffractograms [Fig. 2 ▸(*a*)]. Typically in TEM/SAED, a beamstop hides the primary beam spot and covers some part of the diffraction rings. The resulting absence of a central spot, an asymmetrical diffractogram and abrupt intensity drops around the beamstop distort intensity-based and pixel-wise detection algorithms.

(ii) Diffractograms with broken rings (BR) [Fig. 2 ▸(*b*)]. Common in samples containing mixed micro- and nano­crystals, broken rings are formed by discrete spots at similar radial distances from the center. Although they may appear continuous to the eye, their segmented nature disrupts the rotational symmetry and weakens the radial periodicity, often leading automated methods to lock onto local intensity maxima.

(iii) Reconstruction artifacts (RA) [Fig. 2 ▸(*c*)]. Cross-like patterns centered on the beam spot may arise in 2D diffractograms reconstructed using point spread function deconvolution (*e.g.* from the *STEMDIFF* library; https://pypi.org/project/stemdiff/). While they do not compromise radial averaging or crystallographic interpretation, they bias algorithms that rely on intensity gradients or symmetry, impairing automated detection.

(iv) Strong noise and background (SNB) [Fig. 2 ▸(*d*)]. Aggregating individual patterns introduces a Gaussian-like background with oversaturation and elevated noise near the center, the most critical region for location.

We also identify one further group that is straightforward for center detection, which is rare but close to ideal for 1D profile calculation:

(v) Flat-background (FB) diffractograms [Fig. 2 ▸(*e*)]. There is no Gaussian-like background and diffraction features remain sharp with minimal noise.

We emphasize that these categories are not mutually exclusive. For example, TEM/SAED diffractograms usually include a beamstop and may also exhibit strong noise (Table S2).

#### 4.1.2. Single-crystal diffractograms

In addition to polycrystalline patterns, we evaluated single-crystal patterns because their quality governs the reconstructed composite 2D diffractogram (with Debye–Scherrer rings) and, consequently, the downstream classification. We therefore categorize them as perfect (P) > good (G) > intermediate (I) > bad (B) > worst (W) according to their background/noise levels and peak clarity. Perfect patterns yield clean reconstructions, whereas the worst ones produce noisy diffractograms with barely visible rings (Fig. 3 ▸). As described at the end of Section 2.3, patterns must be categorized on the basis of their observable diffraction characteristics and processing challenges, rather than on sample material alone.

A special subset of single-crystal diffractograms contains a beamstop (Fig. S1). When the spot pattern is approximately centrosymmetric our algorithms can still succeed, but these cases are generally challenging. The beamstop hides the direct beam and its high-intensity neighborhood, as well as disrupting azimuthal information so that the already sparse Bragg spots (compared with continuous rings) exhibit stronger asymmetry and broken periodicity.

### 4.2. Assessment of centering accuracy through radial profiles

**Figure 8:**
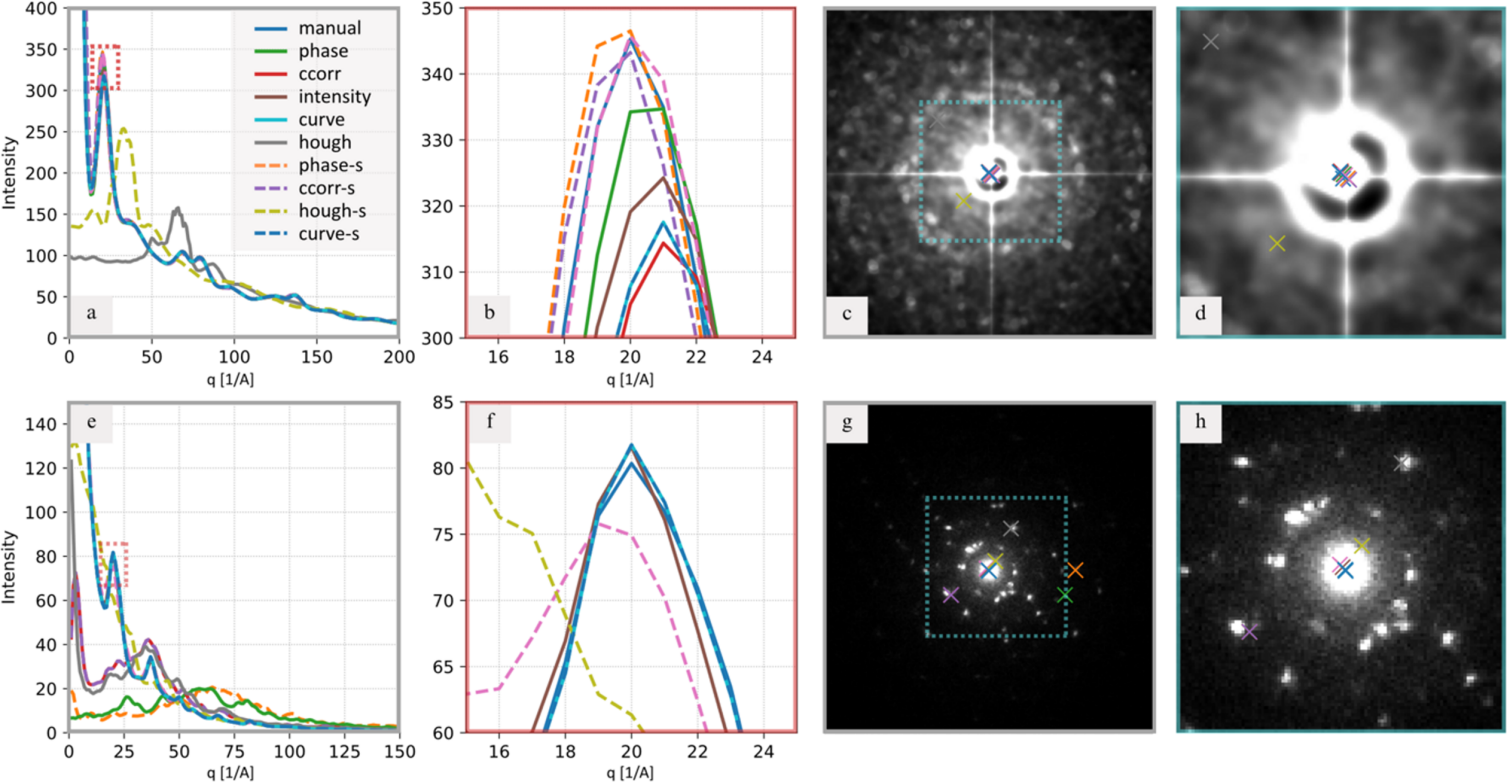
Comparison of detection methods in diffractograms. (*a*) and (*e*) One-dimensional radially averaged profiles of diffractograms calculated from center locations obtained by all detection methods (*manual* = three-point detection, *phase* = phase cross-correlation, *ccorr* = autocorrelation, intensity = maximum intensity detection, *curvefit* = pseudo-Voigt profile fitting and *hough* = Hough transform; methods with an ‘-s’ suffix were applied to edge-detected diffractograms). (*b*) and (*f*) Close-up views of the most intense diffraction peak [highlighted by the red rectangle in panels (*a*) and (*e*), respectively], illustrating how inaccuracies in center location affect the peak’s intensity, location and shape. (*c*) and (*g*) Diffractograms with detected center locations. (*d*) and (*h*) Close-up views of the detected center positions. The samples consisted of (top row) Fe_3_O_4_ nanoclusters with an organic shell using 4D-STEM-in-SEM and (bottom row) Au nanoclusters.

All methods were applied to the available 2D diffraction patterns and their performance could be assessed by analyzing the resulting 1D radially averaged distribution profiles, as illustrated in Fig. 8 ▸. The initial pass – deciding whether a method clearly failed or succeeded – is typically straight­forward: incorrect center location yields visibly distorted or flattened profiles with poorly resolved peaks. A typical example of a poorly suited method for a given diffractogram type is the *hough* method in Fig. 8 ▸(*a*), which yielded a completely wrong 1D profile.

The comparison of the accuracy of those methods that seemed to succeed requires closer inspection. Subtle deviations in center coordinates still degrade peak symmetry, sharpness *etc.* Differences that may seem negligible in the 2D diffractogram [Figs. 8 ▸(*c*), 8 ▸(*d*), 8 ▸(*g*) and 8 ▸(*h*)] become evident in selected parts of the corresponding radial profiles [Figs. 8 ▸(*b*) and 8 ▸(*f*)]: exact peak intensities, sharpness and positions may change, producing a distorted and/or unreliable basis for subsequent crystallographic analysis.

Although discrete well separated diffraction peaks are relatively tolerant to minor center-position errors, the impact is pronounced in regions where weaker peaks are closely spaced or even overlapping. In such cases, especially in the presence of background and noise, peaks may broaden, merge or vanish altogether, undermining the reliability of peak identification.

### 4.3. Detailed comparison of the implemented center detection methods

#### 4.3.1. Polycrystalline diffractograms

For polycrystalline diffraction patterns, the *phase* method proved to be the most universal. The superiority of the *phase* method stems from the fact it operates in the frequency domain and exploits structural symmetry and spatial correlation rather than absolute intensity. The method is tolerant to moderate asymmetry of a diffraction pattern due to a beamstop and is broadly generalizable across acquisition modalities, because the center is inferred from relative pattern similarity rather than local peak characteristics. While other robust spatial-domain approaches, such as sector-wise nonlinear least-squares fitting for low SNR data (Lábár & Das, 2017 ▸), have been successfully applied to particularly challenging diffraction patterns, the *phase* method provides a computationally efficient alternative in the frequency domain that effectively exploits intrinsic structural symmetry.

In contrast, the *intensity* and *curvefit* methods rely on the presence of a discernible central peak; when the region is masked or distorted by a beamstop, the methods are prone to failure. The *ccorr* method approach relies on relatively intact rotational symmetry and periodicity, so broken or discontinuous rings reduce its power. The *hough* method benefits from high-quality continuous ring arcs but remains sensitive to asymmetry and to the missing data blocked by the beamstop.

Preprocessing effects align with these mechanisms: generic edge maps can suppress informative low-frequency content needed by correlation-based methods, whereas *hough* explicitly requires clean edge contours. For this task, Canny’s double-thresholding with hysteresis and Gaussian smoothing yield more reliable ring edges than the Sobel method (Lynn *et al.*, 2021 ▸).

#### 4.3.2. Single-crystal diffractograms

For single-crystal diffraction patterns the situation is more complex due to their lower symmetry, as already discussed above in Section 3. The *phase* approach, which excelled on polycrystalline patterns, assumes symmetric shifts and roughly consistent azimuthal intensity around the center, but single-crystal diffractograms are in general asymmetric, often noisy and/or with high background, which leads to center mis-location. The *ccorr* method depends on recurring structures (such as the concentric rings in powder diffractograms); with isolated diffraction spots it can return false positives because such spots correlate strongly with a central peak template (Liu *et al.*, 2015 ▸; Brewster *et al.*, 2018 ▸). The *hough* detector is ineffective when circular features are absent or fragmentary. Conversely, *intensity* and *curvefit* operate on local intensity structures rather than global symmetry, making them better suited to patterns dominated by the direct beam or discrete Bragg spots. However, the *intensity* method is particularly vulnerable to detector saturation: when the dynamic range of the detector is exceeded near the central beam, the brightest pixels are clipped to the maximum recordable value. This distortion masks the true intensity maximum and can systematically misplace the estimated center, the extent of which also affects the detection: if the area is too small, the true maximum may fall outside the search window, while if it is too large, spurious local maxima or noise can bias the result. Both methods are also sensitive to a poor SNR, where random noise can generate false local maxima or distort intensity gradients, thereby misleading the algorithm and shifting the detected center away from the true position.

The special case of single-crystal diffraction patterns (usually asymmetric) with a beamstop (hiding the central spot) is the most challenging. Fortunately, it is also the rarest case. In the field of TEM, single-crystal diffraction patterns are often aligned by the user along a defined zone axis, which makes them symmetric, and, as a result, the center can be found using the *phase* method, regardless of the beamstop. In the field of 4D-STEM (in both transmission and scanning electron microscopes), the individual diffraction patterns are usually asymmetric, but the pixelated detectors employed in 4D-STEM do not require a beamstop, which makes the center detectable with the *intensity* or *curvefit* methods.

### 4.4. Computational efficiency

Computational efficiency is a critical factor for automated center detection on large datasets where manual annotation is impractical. For conventional diffraction techniques such as TEM/SAED or TEM/NBD, only individual diffractograms are acquired, and a difference of a few fractions of a second when finding the diffractogram center is unimportant. In contrast, modern 4D-STEM experiments generate thousands of diffraction patterns within a single dataset. In such cases, the cumulative computational load becomes substantial, as the center must be detected accurately in each pattern.

While faster methods offer clear time savings, accuracy remains the primary criterion. For example, although the *intensity* method was the fastest, it performed poorly on data featuring beamstops, making slower but more reliable approaches preferable. The *phase* method, despite being slower, consistently achieved the highest accuracy.

The dataset-dependent behavior of *curvefit* suggests sensitivity to data quality: on a dataset with powder diffraction patterns (Fig. 2 ▸), fast but unreliable performance may reflect convergence to local rather than global optima, while on a dataset with single-crystal diffraction patterns (Fig. 3 ▸) the method was slower yet more accurate, probably because clearer peak profiles allowed more effective parameter-space exploration. Similarly, the increase in runtime for the *hough* method coupled with Canny pre-processing can be attributed to the suppression of weaker edges, reducing the feature density and prolonging voting and peak selection.

#### 4.4.1. Practical guide: when to use which method

We distilled our results into a practical guide for selecting a center-detection method (Table 5 ▸). The decision table maps common pattern characteristics to recommend reliable algorithms. Generally, *ccorr* should be avoided: it failed nearly completely on monocrystals and was inconsistent on polycrystalline diffractograms. *phase* is the default for 2D polycrystalline diffractograms with Debye–Scherrer rings; *curvefit* (and, where appropriate, *intensity*) suits single-crystal frames dominated by discrete diffraction spots.

#### 4.4.2. Applicability of our software to X-ray, neutron and light diffraction

Our software focuses on electron diffractograms, but the underlying requirement – accurate location of the direct beam in 2D diffraction images – extends well beyond electron diffraction. As summarized in Table 6 ▸, similar demands are reported in other types of diffraction, where 2D detectors are occasionally used. Regardless of the diffraction type, the diffraction patterns are similar in the sense that they contain diffraction rings for powder-like samples and individual diffraction spots for monocrystal-like samples.

## 5. Conclusions

This work targets accurate fully automatic center location in 2D electron diffraction patterns. We have implemented six center detection and three center refinement algorithms in Python and released them in ediff.center, a module of the open-source *EDIFF* package (https://pypi.org/project/ediff). All methods were evaluated systematically on a representative suite of diffractograms – single crystal and polycrystalline, with and without a beamstop, and spanning a range of background and/or noise levels. The main conclusions are:

(i) The algorithms in ediff.center can locate the center of all common 2D electron diffractograms in a straightforward automated manner. From the user’s perspective, it suffices to select and call a function appropriate to the pattern type.

(ii) Center detection is generally easier in polycrystalline diffractograms due to their higher radial symmetry (Debye–Scherrer rings). The most universal automatic method is *phase* (phase cross-correlation exploiting inversion symmetry), followed by *hough* (circular feature detection using the Hough transform).

(iii) Single-crystal diffractograms are more challenging. In modern 4D-STEM data, a strong direct beam spot often allows accurate location using the *curvefit* and *intensity* methods, which target the central peak. In traditional TEM/NBD experiments, diffractograms are usually aligned along some specific user-defined zone axis, which makes them symmetric. The center can be determined using the *phase* method as in symmetric polycrystalline patterns, even if the central spot is hidden by a beamstop.

Overall, our module ediff.center offers a general modality-agnostic solution for center determination in diffraction data; the same principles extend to X-ray, neutron and light diffraction.

## 6. Related literature

For further literature related to the supporting information, see Bücker *et al.* (2021 ▸), Filik *et al.* (2017 ▸), Hammersley (2016 ▸), Heybrock *et al.* (2025 ▸), Kieffer & Karkoulis (2013 ▸), Mitchell (2008 ▸) and Prescher & Prakapenka (2015 ▸).

## Supplementary Material

Additional details plus extra figures and tables. DOI: 10.1107/S1600576726002384/hat5018sup1.pdf

## Figures and Tables

**Table 1:** Datasets for testing center detection methods

Dataset	No.	Description	Details
D1	15	Polycrystalline diffraction patterns exhibiting circular patterns, acquired either via TEM/SAED or 4D-STEM-in-SEM	Table S2
D2	75	Single-crystal diffraction patterns, acquired via 4D-STEM-in-SEM	Table S3

**Table 2:** List of center detection methods

Method	Brief description†
*manual*	User clicks three ring points and the center is triangulated
*intensity*	Calculates maximum intensity within a central region
*curvefit*	Fits a pseudo-Voigt profile to the central peak
*phase*	Phase cross-correlation detection of inversion symmetry
*ccor*	Maximum autocorrelation peak
*hough*	Detection of circular features using Hough transform
*none*	No center detection, the center coordinates are inserted or read from a file

† Detailed descriptions of all methods are given in the supporting information, Section S1.2.

**Table 3:** List of center refinement methods

Method	Brief description
*manual*	Manual refinement – shifts of overlaid diffraction ring using keyboard controls
*sum*	Radial intensity sum maximization – auto-shifts of overlaid diffraction ring
*var*	Radial intensity variance minimization – auto-shifts of overlaid diffraction ring
*none*	No refinement – the coordinates from the center detection step are unchanged

**Table 4:** Classification of ED patterns

Class	Description	Sample
BB	Beamstop blocks primary beam	Polycrystalline (rings)
BR	Broken rings (incomplete Debye–Scherrer rings)	Polycrystalline (rings)
RA	Reconstruction of artifacts from deconvolution	Polycrystalline (rings)
SNB	Strong noise and background	Polycrystalline (rings)
FB	Flat (ideal) background	Polycrystalline (rings)
P	Perfect quality	Single crystal (spots)
G	Good quality	Single crystal (spots)
I	Intermediate quality	Single crystal (spots)
B	Bad quality	Single crystal (spots)
W	Worst quality	Single crystal (spots)

BB, BR, RA, SNB and FB are diffractogram classifications based on image features. P, G, I, B and W are diffractogram classifications based on image quality in terms of noise and background levels. In Fig. 2 there are typical diffractograms from each category.

**Table 5:** Practical guide to determine when to use which center detection method

Dataset or category†	Primary method	Why	Fallback	Notes
Polycrystalline (D1)	*phase*	Robust to intensity fluctuations and occlusions; uses symmetry and correlation	*hough* or *curvefit*	The best overall on D1, subpixel accuracy (see Figs. 3 and 4)
BB	*phase*	Tolerates missing data	*hough*	The implementation uses an explicit beamstop mask
BR	*curvefit*	Models discrete spots/arc segments	*phase*	*hough* if arcs are long/clean
RA	*phase*	Uses symmetry and correlation	*ccorr*	Edge detection prior to center determination is beneficial in this case
SNB	*phase*	Less reliant on absolute intensity	*curvefit*	Requires background normalization
FB	*phase*	Strongest/sharpest peaks	Any	An easy case, multiple methods are applicable
Single-crystal (D2)	*curvefit*	Works on local peak/spot structure	*intensity*	No automatic method beats the reference; *manual* is still the best

† Category tags are according to the classifications in Table 4.

**Table 6:** Diffraction techniques employing 2D detectors The final column gives brief comments concerning the 2D detectors employed by the given diffraction method.

Diffraction method	Radiation type	Typical 2D detectors	Comments
Electron diffraction	Electrons	CCD/CMOS, hybrid-pixel	Area detectors are standard in TEM/STEM setups (Heidler *et al.*, 2019 ▸; Slouf *et al.*, 2021*a* ▸)
X-ray diffraction	X-rays	CCD/CMOS, hybrid-pixel	Point detectors are perhaps more common, but area detectors for recording 2D diffractograms are used as well (Ashiotis *et al.*, 2015 ▸)
Neutron diffraction	Neutrons	2D area detectors (*e.g.* image plates, pixel detectors)	Increasingly employs pixelated detectors, especially in time-of-flight setups (Jäger *et al.*, 2024 ▸), with beam center finding required for data reduction
Light diffraction	Visible or NIR light	CCD/CMOS, photodiode arrays	2D detectors used in laser diffraction and holographic methods (Siavashani *et al.*, 2021 ▸); 2D fringe or ring patterns require identification of the central spot

## Data Availability

All diffraction patterns, calculations and results from this work are available from the first author (pavlinasik@isibrno.cz) upon request. The *EDIFF* Python package that contains the ediff.center module with all algorithms employed in this study is freely available in the standard PyPI repository (Python Package Index): https://pypi.org/project/ediff.
