# Supplementary material for: Computational methods for automated center determination in electron diffraction patterns
Source: J Appl Crystallogr. 2026 Apr 30;59(Pt 3):845–57. doi: 10.1107/S1600576726002384 (PMC13224791; doi:10.1107/S1600576726002384)
Supplement: Supplementary file 1 [file j-59-00845-sup1.pdf]

JOURNAL OF APPLIED CRYSTALLOGRAPHY

**Volume 59 (2026)**

**Supporting information for article:**

# Computational methods for automated center determination in electron diffraction patterns

**Pavlina Sikorova, Miroslav Slouf, Tomas Molnar and Vladislav Krzyzanek**

# S1. Supplementary text

## S1.1. EDIFF: ellipticity correction

The package EDIFF now already provides a tool for ellipse correction (*ediff.center.ellipse_distortion*), which warps four manually selected points (ideally located on a distorted ring) into positions that form a perfect circle. This is especially useful for correcting geometrical distortions in diffractograms caused by acquisition conditions.

Even though this is typically not a problem of 4D-STEM-in-SEM nor TEM/SAED data, other diffraction-based techniques, such as low-energy electron microscopy (LEEM), may more commonly suffer from this artifact—and for further analysis, it is necessary to correct this artefact.

Since LEEM operates at energies below 100 eV, it is highly surface-sensitive, with a typical probing depth of only a few atomic layers. This high surface sensitivity enables the study of surface atomic reconstructions, which can then be correlated with models of the actual atomic positions. If the sample or electron beam is imperfectly aligned, the typical circular, centrosymmetric diffraction pattern may become distorted into an ellipse (see Figure S2). Correcting this distortion allows for a more accurate representation of the data when modelling the surface atomic structure. Within the frame of this work, the ellipticity correction improves the quality of radially averaged profiles, enhancing the performance of the EDIFF library.

Currently, the correction is semi-automatic, as the user must manually select diffraction spots along the distorted ring. Each point is refined to the maximum local intensity to ensure accurate alignment. The function calculated the geometric center and average radius of the distorted quadrilateral and maps it to a circle using a homography transformation (a projective transformation generalizing affine transformations by allowing perspective distortion correction). The image is then transformed using this homography, with additional padding to ensure the output is square and centered (for the sake of compatibility with other EDIFF tools). This approach ensures a more isotropic circular shape.

## S1.2. Methods for center detection

In this section, the methods implemented for detecting the center in diffractograms are described. Methods vary in their level of automation, sensitivity to artifacts, and applicability to different types of diffraction patterns. The goal is to provide flexible options depending on image properties.

### S1.2.1. Three-point detection (*manual*)

The first implemented method is the only one that is not fully automated, but its strength is its universal applicability. The user must identify three pixels that lie within a diffraction circle in the image. Then, the circle is calculated based on the geometric concept of circumcenter of a triangle. Following

this initial center calculation, the user has the option to refine the center coordinates using keyboard arrows if necessary (summarized workflow in Figure S3).

### S1.2.2. Maximum intensity detection (*intensity)*

A small region around the image center is extracted, where the first central moments are calculated. Those are then used to compute the centroid in the central region which serves as the output coordinates. In Figure S4, there is a respective block-scheme.

### S1.2.3. Phase cross-correlation detection (*phase*)

This approach is based on locating the center of symmetry in a diffraction image using a combination of weighted intensity averaging and phase cross-correlation (René De Cotret *et al.*, 2018). There are two crucial steps: (i) estimation of the initial center using weighted averaging of pixel intensities, and (ii) refinement of the center position by analyzing inversion symmetry through phase cross-correlation. After obtaining the center estimate, the image is cropped around its position, as the central region is the region of interest (not all diffraction patterns are centered). Intensity variations due to experimental conditions are suppressed by background normalization. The phase cross-correlation is calculated between the cropped region and its inverted copy. Fourier transform is applied to both, and their cross-power spectrum is calculated to obtain their phase shift. The inverse Fourier transform translates the phase shift back, which converts it to a spatial shift (Δx and Δy increments to the initial center location estimation), revealing how far the images are offset from each. The whole workflow is summarized in Figure S5, and a specific example is shown in Figure S6 and Figure S7.

### S1.2.4. Autocorrelation detection (*ccorr*)

The primary distinction between this method and phase cross-correlation lies in the domain in which the correlation is calculated—here, we estimate the center position from the spatial-domain correlation of two images. Therefore, the workflow remains identical until the generation of the inverted copy (template) of the processed original image (source). The template is cropped down to a quarter of its original size and then systematically translated over the source in a pixel-wise manner. At each position, the correlation between the overlapping pixels is calculated, resulting in a 2D correlation matrix, where each element represents the degree of symmetry. The location of the maximum correlation coefficient in this matrix corresponds to the position where the template aligns best with the original, which is taken as the center of symmetry. The workflow's block-scheme is summarized in Figure S8, and the intermediate results of this method are shown in Figure S9.

### S1.2.5. Hough transform detection (*hough*)

This method (block scheme in Figure S10) can be applied to real diffraction data that exhibit circular diffraction patterns (example in Figure S11). The first step involves image preprocessing to ensure that

edges (circles) are clearly distinguishable from the background. According to (Adatrao & Mittal, 2016), who studied multiple edge detection techniques prior to CHT, various approaches exist for this task. Within our Python package, we employed the Canny edge detector and Sobel filter. A search space for circles is defined, and the Hough transform is applied to each. Peaks in the Hough space are identified—if a peak is found, the corresponding circle's center and radius are stored. After processing the entire search space, the final circle is selected based on the highest accumulated value in Hough space, and the detected circle's center and radius is recorded.

#### S1.2.6. Pseudo-Voigt profile fitting detection (*curvefit*)

If we fit a 2D pseudo-Voigt model to the central region of the image, we may estimate the center of a diffraction pattern. The model is well suited to describe symmetric and radially decaying intensity peaks, which are commonly found at the center of diffractograms.

The algorithm begins by assuming the geometric center of the image as the approximate diffractogram center location. A square region (ROI) is extracted around this point to isolate the central peak and omit possible misleading peaks. The fitting function is a 2D pseudo-Voigt profile defined by six parameters (amplitude, [x, y] center coordinates, standard deviations along x, y, and the mixing ratio between Gaussian and Lorentzian components). The model is fitted to the observed intensity distribution in the ROI, minimizing the difference between the model and actual data. The refined [x, y] coordinates obtained from the fit are converted back to full-image coordinates. Figure S12 shows the whole workflow of this automatic detection method.

### S1.3. Influence of beamstop presence on center detection performance in monocrystalline diffraction patterns

The presence or absence of a beam stopper strongly influences the performance of center-detection algorithms in monocrystalline diffraction patterns. In the absence of a beam stopper, the central beam dominates the intensity profile, allowing manual and intensity-based methods to precisely identify the pattern center, as demonstrated in this work. However, this same dominance renders the phase-correlation method less effective, since the overwhelming low-frequency signal can distort phase information. Conversely, when a beam stopper obscures the direct beam, the intensity distribution becomes more balanced, allowing phase correlation to exploit the symmetric arrangement of diffraction features to accurately locate the geometric center.

Another factor influencing the performance of center-detection methods in monocrystalline diffractograms with beam stoppers is the structural regularity and symmetry of the diffraction pattern. When the discrete Bragg reflections are evenly distributed around the true center, the phase-correlation method provides reliable results. However, in cases of irregular or asymmetric spot distribution, manual center identification remains the more dependable approach.

## S2. Supplementary figures

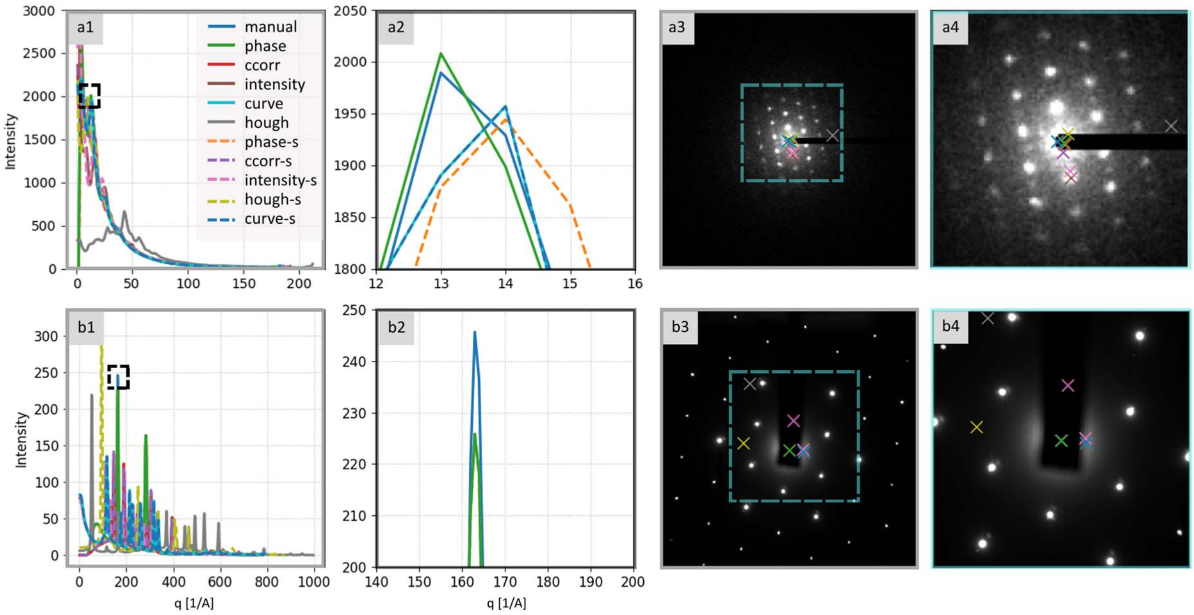


**Figure S1** **Performance of center detection methods on representative monocrystal diffractograms with a beamstop.** Column 1: 1D radially averaged profiles computed using each method's center (overlaid, color-coded). Column 2: zoom of the dominant peak (black box in 1), showing the top-performing method(s). Column 3: original diffractogram with detected centers (color-coded crosses). Column 4: zoom of the central region (blue box in 3) around the direct (primary) beam and the detections. Samples: (a) $TbF_3$ nanocrystalline aggregates with simulated beamstopper, (b) graphite from (René De Cotret *et al.*, 2018) .

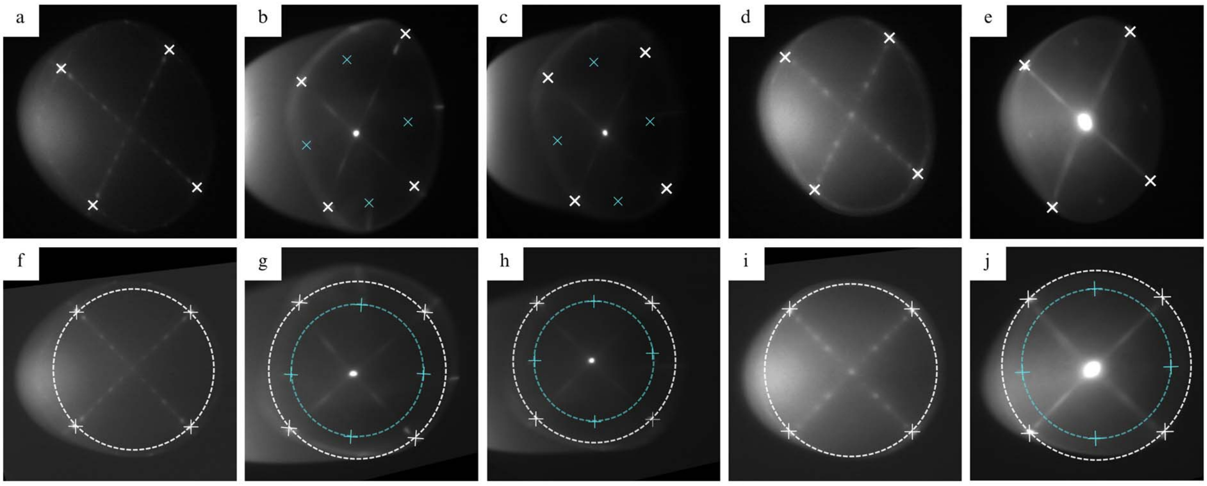


**Figure S2** **Demonstration of elliptical distortion correction in LEEM data using the ediff.center module.** (a—e) Original distorted diffractogram with manually selected points used to compute the affine transformation. (f—j) Corrected diffractogram after transformation, with visualization of the diffraction spots, showing their radial positions relative to the center, confirming restored circular symmetry. Sample: epitaxial FeRh thin film.

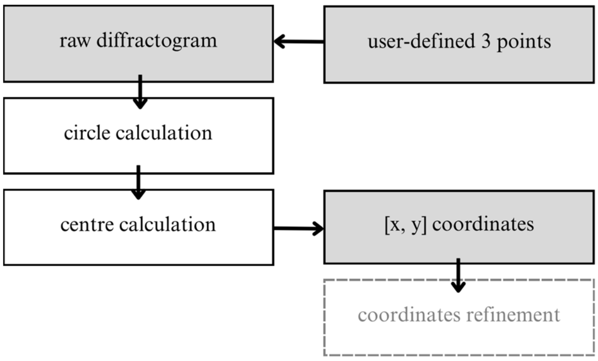


**Figure S3** **Workflow for interactive center detection.** The user manually selects three points that lie on a circular diffraction ring within the image. The center is then computed as the circumcenter of the triangle formed by these three points. If the selected points were slightly inaccurate, the estimated center can be manually adjusted to refine its position in the end.

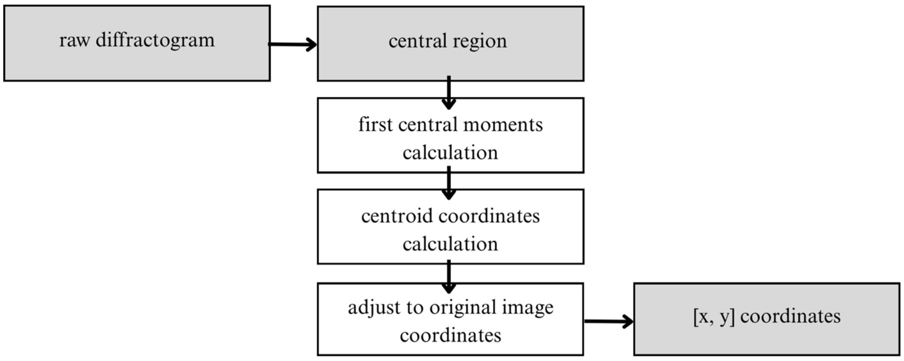


**Figure S4 Workflow for maximum intensity detection.** From the raw diffractogram, a small central region is extracted. There, the first central moments are calculated, and they are used to compute the centroid of the region. The centroid's coordinates are then translated back to the dimensions of the original diffractogram.

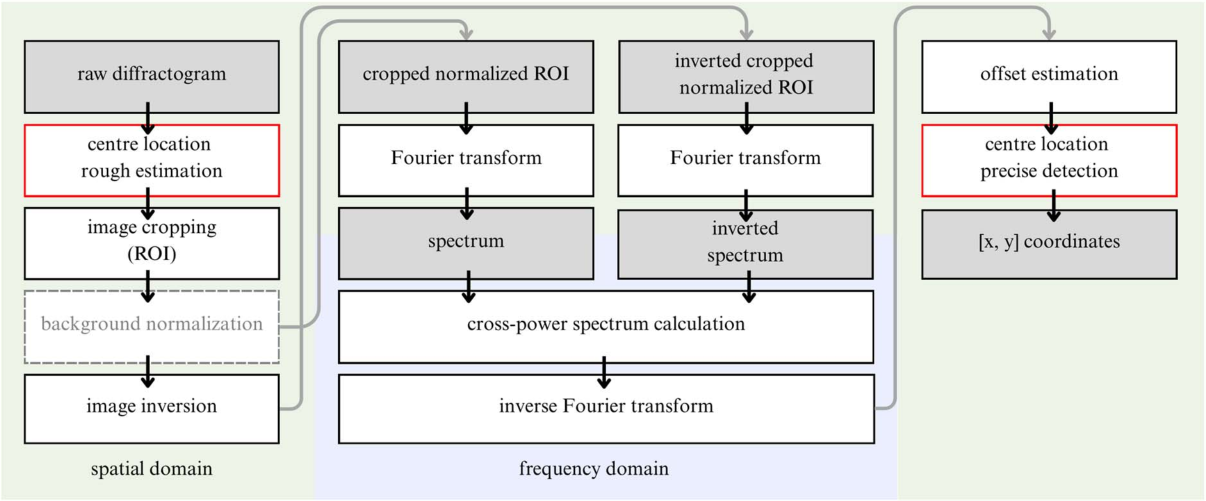


**Figure S5 Workflow for phase cross-correlation center detection**. The raw diffractogram is first processed in the spatial domain (green background): initial estimation of the center position, selection of the region of interest and subsequent normalization. Then the inverted counterpart is generated. Both images undergo Fourier transform that moves the operations into the frequency domain (blue background). Their cross-power spectrum is calculated to preserve the phase shift information. Inverse Fourier transform then maps phase shifts to spatial offsets, which refines the center location and allows accurate center detection.

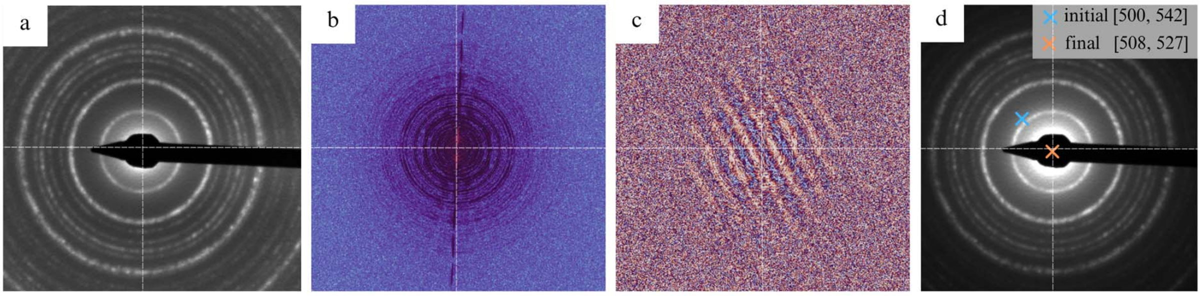


**Figure S6 Intermediate results of phase cross-correlation detection method.** Image (a) shows the processed diffractogram in the spatial domain. The Fourier spectra of (a) is presented in (b). The cross-power spectrum (CPS) of the (b) and spectra of inverted copy of (a) is in (c), and center detection is visualized in (d). The CPS in (c) should resemble a sharp peak at the center if the images are well aligned. Here, the peak is spread around the center, which indicates an offset between image and its inverted counterpart. Sample: light-upconverting $NaYF_4$ nanocrystals.

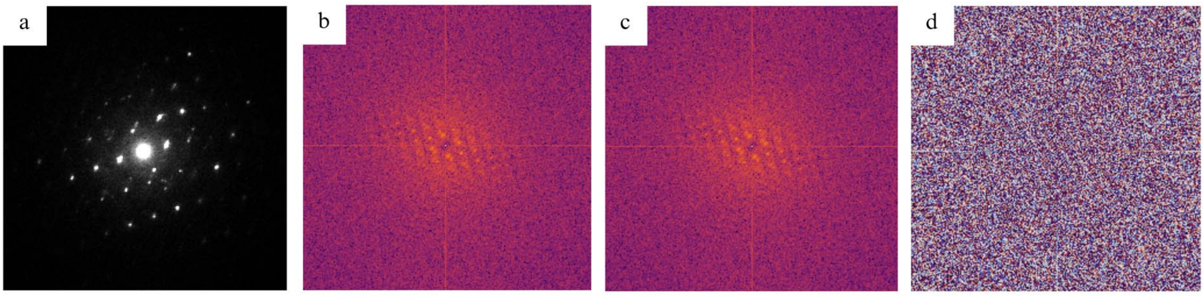


**Figure S7 Intermediate results of phase cross-correlation detection method.** Image (a) shows original input diffraction pattern in the spatial domain. The Fourier spectra of (a) and its inverted copy are in (b) and (c), respectively. The cross-power spectrum (CPS) is in (d). The CPS should resemble a sharp peak at the center if the images are well aligned, which would identify the center position in the geometrical center of the image. If images were misaligned, the peak would spread around the center and still carrying information about the shift in spatial domain—however in the case of Bragg spots, no peak is distinguishable, which prevents the method from detecting any changes in phase domain that could be converted to spatial domain. Therefore, the phase cross-correlation method fails in the case of center detection of individual diffraction patterns from 4D-STEM-in-SEM. Sample: $Fe_3O_4$ nanoclusters from 4D-STEM/PNBD experiment (Slouf *et al.*, 2025).

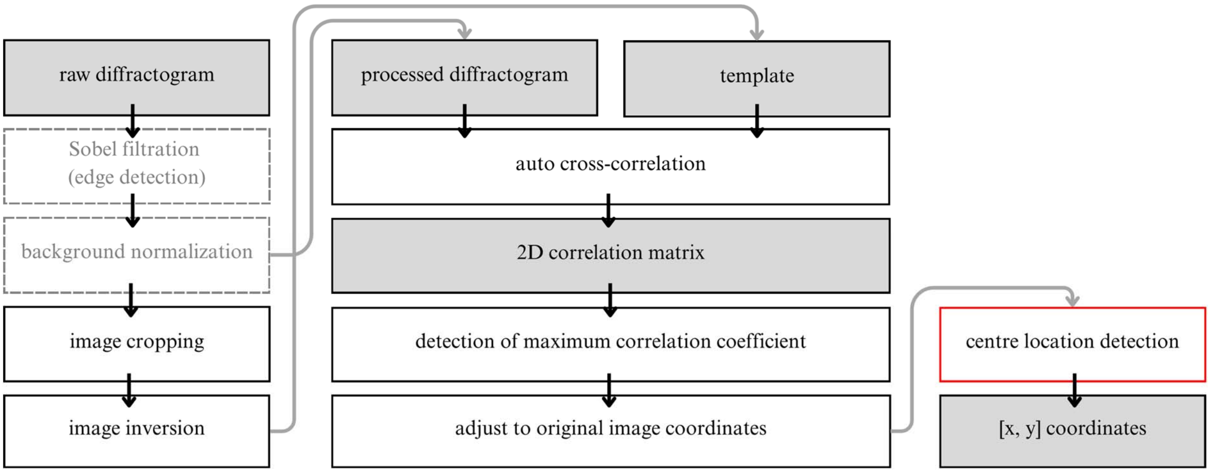


**Figure S8** **Workflow for automated center detection using autocorrelation.** Starting from the raw image, optional Sobel filtering and background normalization are applied for pre-processing (processed diffractogram). A central region is then cropped and inverted (template). Cross-correlation between the processed diffractogram and template yields a 2D correlation matrix. The location of the maximum correlation coefficient is identified and mapped back to the original coordinate system to determine the true center position.

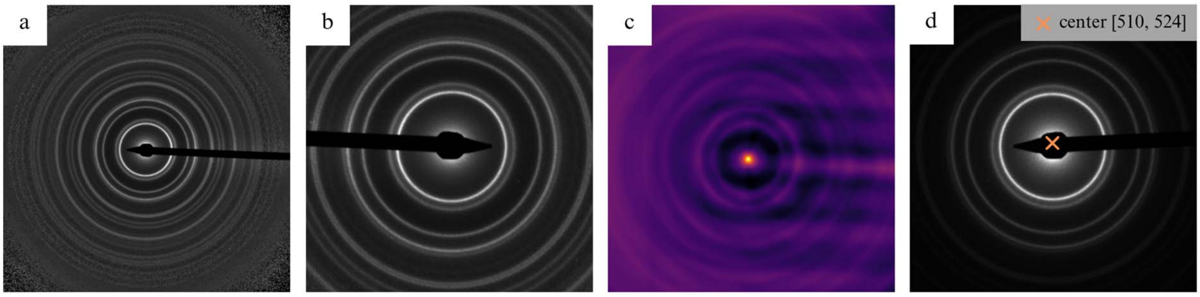


**Figure S9** **Intermediate results of autocorrelation detection method.** Image (a) shows the pre-processed diffractogram (source image). The template (b), obtained by cropping and inverting the central region of the respective source image, is systematically shifted across the source image to compute a correlation coefficient at each position. This results in a normalized cross-correlation matrix (c) that reflects the degree of local inversion symmetry in each element, where the brightest point (highest correlation coefficient) marks the diffractogram center. The final center location (d) is determined by converting this peak location back to the coordinate system of the original image and is highlighted as an orange marker. Sample: Au nanoislands, diffraction patterns from TEM/SAED.

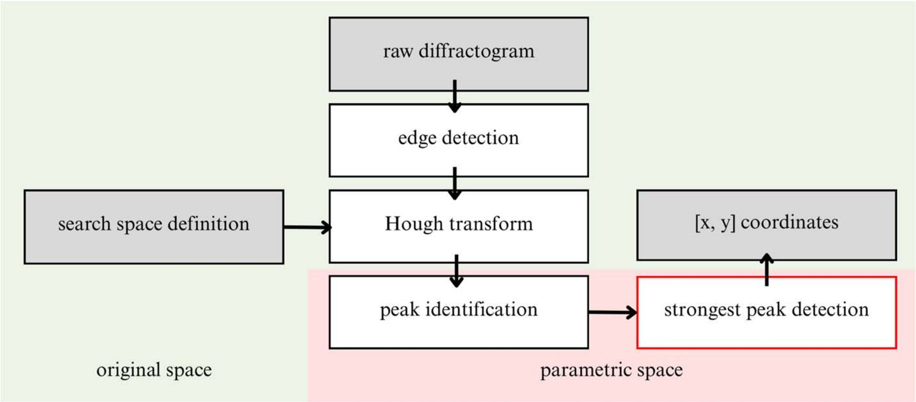


**Figure S10Workflow for automated center detection using Hough transform.** In the raw diffractogram, edges are detected using either the Canny detector or the Sobel operator. The resulting edge-image is then mapped into a 3D parametric space (defined by the center coordinates and radius), using the Circular Hough transform (CHT). This transformation is constrained by a predefined search space that specifies minimum and maximum radius range. The resulting accumulator space encodes the likelihood of circle presence at each point, with higher intensities corresponding to stronger evidence for a circle centered at those coordinates and with a given radius. Peaks in the accumulator space are identified, and the peak with the highest intensity is selected as the most probable center of the diffraction pattern.

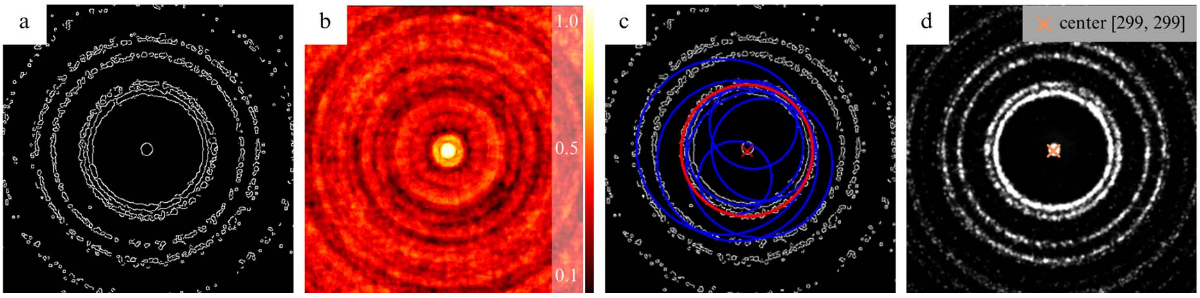


**Figure S11Intermediate results of Hough detection method.** (a) Diffractogram after filtering with the Canny edge detector, (b) corresponding Hough parameter space, where intensity indicates the likelihood of a center position (refer to the color scale adjacent to the images). (c) Detected circles in the original images using the Hough transform (blue: all detected circles; red: selected circle corresponding to the final center). (d) Final estimated center positions based on the selected red circle. Sample: Au nanoislands, diffraction patterns from 4D-STEM/PNBD experiment (Slouf *et al.*, 2025).

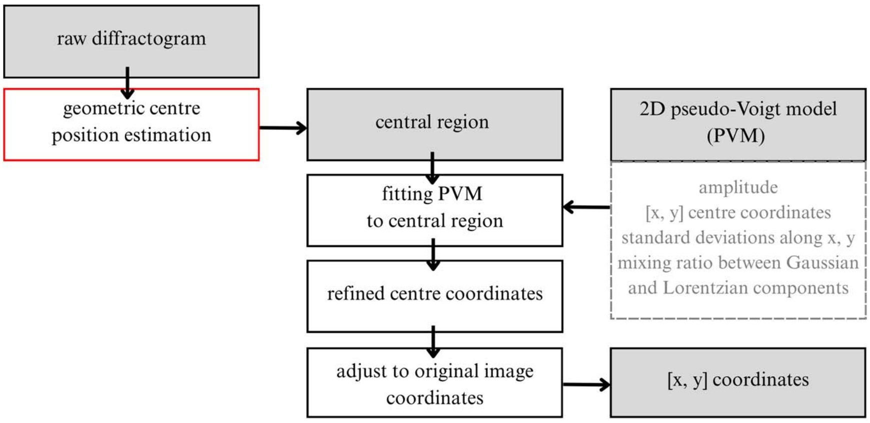


**Figure S12 Workflow for pseudo-Voigt profile fitting detection.** A square region of interest (ROI) is extracted, and initial center position is estimated. The fitting function PVM is defined by six parameters (amplitude, [x, y] center coordinates, standard deviations along x, y, mixing ratio between Gaussian and Lorentzian components). PVM is fitted to the observed intensity distribution in the ROI, minimizing the difference between the model and actual data.

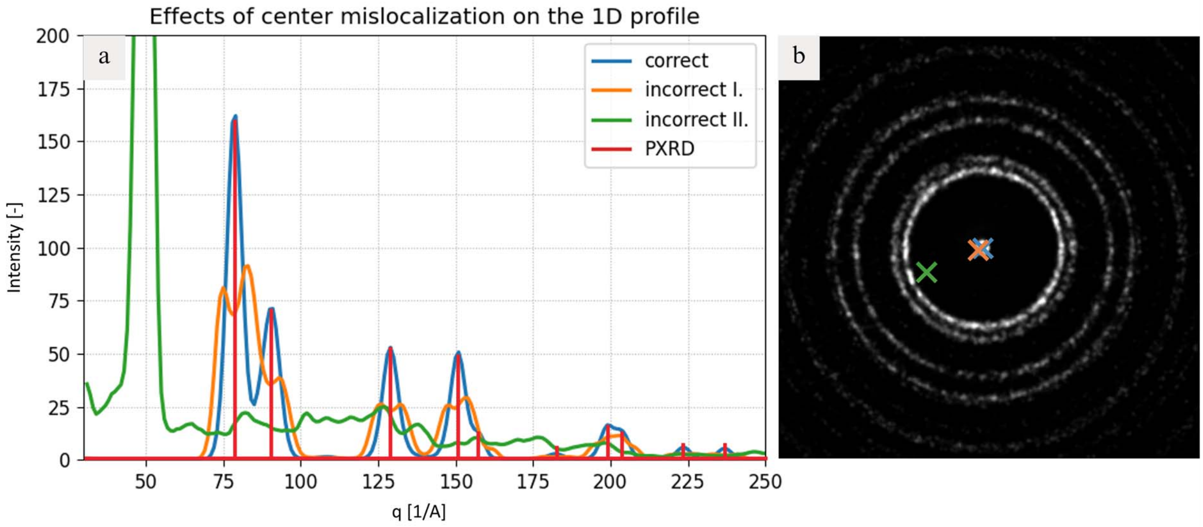


**Figure S13 Effect of incorrect localization of the diffraction pattern center on the 1D radially averaged intensity profile.** (a) radially averaged intensity profiles calculated using the correctly determined center (blue), two intentionally mislocalized centers (orange and green), and the theoretical powder XRD reference calculated from a .cif file (red). Even small deviations from the true center lead to peak broadening and splitting, intensity redistribution, and shifts in apparent peak positions, whereas larger offsets significantly distort the profile and suppress peak contrast. (b) representative diffraction pattern with the correctly determined center (blue cross) and two mislocalized center positions (orange and green crosses) overlaid. The displacement of the assumed center directly alters the radial geometry, resulting int the profile distortions observed in panel (a).

## S3. Supplementary tables

**Table S1** **SW featuring center detection in diffractograms (sorted from the newest to oldest)**

| SW | Reference | Diffraction | Availability | Center detection |
|---|---|---|---|---|
| **EDIFF** | (Slouf *et al.*, 2025) | any | Python, open source | multiple (see this article) |
| **ESS Scipp SANS beam-center finder** | (Simon Heybrock *et al.*, 2025) | neutron | Python, open source | center-of-mass |
| **pyxem** | (Cautaerts *et al.*, 2022) | 4D-STEM | Python, open source | cross correlation, center-of-mass, lattice-fitting |
| **py4DSTEM** | (Savitzky *et al.*, 2021) | 4D STEM | Python, open source | cross correlation, lattice-fitting |
| **diffractem** | (Bücker *et al.*, 2021) | X-ray | Python, open source | center-of-mass, Lorentzian function fitting |
| **LiberTEM** | (Clausen *et al.*, 2020) | 4D-STEM | Python, open source | center-of-mass, lattice-fitting |
| **Scikit-ued** | (René De Cotret *et al.*, 2018) | TEM/SAED | Python, open source | phase cross-correlation |
| **DAWN2** | (Filik *et al.*, 2017) | X-ray | Java, open source | manual, Gaussian function fitting |
| **FIT2D** | (Hammersley, 2016) | X-ray | Fortran90/ANSI-C, free | Gaussian function fitting |
| **CrysTBox** | (Klinger & Jäger, 2015) | TEM/SAED | MATLAB, closed source | Hough transform |
| **DIOPTAS** | (Prescher & Prakapenka, 2015) | X-ray | Python, open source | powder calibrant ring fitting |
| **pyFAI** | (Kieffer & Karkoulis, 2013) | X-ray | Python, open source | Geometry/center refinement from Debye-Scherrer rings |
| **DigitalMicrograph** | (Mitchell, 2008) | TEM/SAED | closed source | Hough transform |
| **Process Diffraction** | (Lábár JL, 2000) | any | free | manual |

**Table S2** **Overview of polycrystalline diffractograms analyzed (dataset D1)**

| ID | Sample | Description | Acquisition | Class | Type |
|---|---|---|---|---|---|
| 1 | $NaYF_4$ | strong and sharp diffractions | TEM/SAED | 1 | beamstop-blocked |
| 2 | Au | nanoparticles, very strong diffractions, lower background | TEM/SAED | 1<br>5 | beamstop-blocked,<br>flat background |
| 3 | $NaYF_4$ | strong and sharp diffractions | TEM/SAED | 1<br>4 | beamstop-blocked,<br>strong noise/background |
| 4 | $Fe_3O_4$ | weak diffractions | TEM/SAED | 1<br>4 | beamstop-blocked,<br>strong noise/background |
| 5 | ZnO-CuO | very weak diffractions | TEM/SAED | 1<br>4 | beamstop-blocked,<br>strong noise/background |
| 6 | Ag | mixture of micro- and nanocrystals | TEM/SAED | 1<br>2 | beamstop-blocked,<br>broken rings |
| 7 | Ag | mixture of micro- and nanocrystals | TEM/SAED | 1<br>2 | beamstop-blocked,<br>broken rings |
| 8 | Au | strong diffractions | 4D-STEM/PNBD | 5 | flat background |
| 9 | $Fe_3O_4$ | clusters, weak diffractions | 4D-STEM/PNBD | 3<br>4 | reconstruction artifacts,<br>strong noise/background |
| 10 | $Fe_3O_4$ | strong diffractions | TEM/SAED | 1<br>4 | beamstop-blocked,<br>strong noise/background |
| 11 | $NaYF_4$ | strong and sharp diffractions | TEM/SAED | 1 | beamstop-blocked |
| 12 | $NaYF_4$ | strong and sharp diffractions | TEM/SAED | 1 | beamstop-blocked |
| 13 | $TbF_3$ | strong and blurry diffractions | 4D-STEM/PNBD | 3<br>4 | reconstruction artifacts,<br>strong noise/background |
| 14 | $LaF_3$ | very weak diffractions | 4D-STEM/PNBD | 3<br>4 | reconstruction artifacts,<br>strong noise/background |
| 15 | $GdF_3$ | strong and blurry diffractions | 4D-STEM/PNBD | 3<br>4 | reconstruction artifacts,<br>strong noise/background |

**Table S3** **Overview of analyzed monocrystal patterns (dataset D2).** They are categorized as *perfect* > *good* > *intermediate* > *bad* > *worst* based on background/noise levels and peak clarity.

| ID | Sample | Description | Class | Quality |
|---|---|---|---|---|
| 01—15 | Au | nanoparticles, very strong diffractions, low background | 1 | perfect |
| 16—30 | $TbF_3$ | nanocrystalline aggregates, strong diffraction, high background | 2 | good |
| 61—75 | $GdF_3$ | nanocrystalline aggregates, strong and blurry diffraction, high background | 3 | intermediate |
| 31—45 | $Fe_3O_4$ | nanoclusters, weak diffractions, high background | 4 | bad |
| 46—60 | $LaF_3$ | nanoparticles, very weak diffractions, very high background | 5 | poor |

**Table S4** **Average execution time for each detection method on both datasets D1 and D2.** Methods (*phase* = phase cross-correlation, *ccorr* = autocorrelation, *intensity* = maximum intensity detection, *curvefit* = pseudo-Voigt profile fitting, *hough* = Hough transform) were tested with (A) and without (B) image preprocessing. D1 = polycrystalline dataset, D2 = monocrystal dataset

| Detection method | Mean time [s] | | Median [s] | | Standard deviation [s] | |
|---|---|---|---|---|---|---|
| | D1 | D2 | D1 | D2 | D1 | D2 |
| *phase* (A) | 2.431 | 0.196 | 1.43 | 0.186 | 1.78 | 0.035 |
| *phase* (B) | 2.509 | 0.198 | 1.38 | 0.191 | 1.96 | 0.025 |
| *ccorr* (A) | 0.232 | 0.009 | 0.12 | 0.010 | 0.20 | 0.006 |
| *ccorr* (B) | 0.289 | 0.012 | 0.14 | 0.012 | 0.23 | 0.005 |
| *intensity* (A) | 0.022 | 0.001 | 0.02 | 0.001 | 0.03 | 0.002 |
| *intensity* (B) | 0.066 | 0.002 | 0.04 | 0.002 | 0.05 | 0.003 |
| *curvefit* (A) | 0.140 | 11.957 | 0.12 | 11.454 | 0.08 | 0.648 |
| *curvefit* (B) | 0.145 | 12.081 | 0.11 | 11.324 | 0.09 | 0.477 |
| *hough* (A) | 34.681 | 1.923 | 1.94 | 1.884 | 73.28 | 0.648 |
| *hough* (B) | 60.032 | 1.495 | 44.81 | 1.440 | 54.23 | 0.477 |

Tests were conducted on a system with the following specifications:13th Gen Intel® Core™ i7-1355U CPU @ 1.70 GHz (turbo up to ~5.0 GHz), 32 GB RAM, 64-bit Windows operating system with 12 cores. The code was executed using Python 3.10 with NumPy/SciPy on a standard laptop configuration.
